# Tracheotomie: Indikation und Durchführung

**DOI:** 10.1007/s00063-024-01184-2

**Published:** 2024-10-11

**Authors:** Michael Oppert, Markus Jungehülsing, Lutz Nibbe

**Affiliations:** 1 Zentrum für Intensiv- und Notfallmedizin, Klinikum Ernst von Bergmann gGmbH, Charlottenstraße 71, 14467 Potsdam, Deutschland; 2 Klinik für Hals‑, Nasen‑, Ohrenheilkunde, Klinikum Ernst von Bergmann gGmbH, Potsdam, Deutschland; 3 Health and Medical University, Potsdam, Deutschland

**Keywords:** Atemwegsmanagement, Beatmung, Notfallmedizin, Komplikationen, Schluckstörungen, Airway management, Pulmonary ventilation, Emergency care, Complications, Swallowing disorders

## Abstract

Die Tracheotomie wird bereits seit langer Zeit außerhalb der Intensivmedizin durchgeführt. In der modernen Medizin hat sie einen festen Platz in der Therapie von Intensiv- und Notfallpatienten, aber auch in der Tumorchirurgie des Kopfs und des Halses, der Versorgung langzeitbeatmeter Patienten, bei Patienten mit Schluckstörungen und neurologischen Erkrankungen. Entsprechend den unterschiedlichen Erkrankungen sind Indikation, Durchführungstechnik und Versorgung mit Kanülen sowie die Langzeitbetreuung sehr unterschiedlich; dieser Artikel bietet eine Übersicht über die unterschiedlichen Indikationen und Operationstechniken und diskutiert den optimalen Zeitpunkt einer Tracheotomie in der modernen Intensivmedizin.

## Abstract (en)

Tracheotomy has long been performed outside of intensive care medicine. In modern medicine, it has a firm place in the management of critically ill and emergency care patients as well as in cancer surgery of the head and neck, the care of long-term ventilated patients, patients with swallowing disorders, and neurological diseases. The indication, technique, and timing of tracheotomy are very different for the various diseases. This article provides an overview of the different indications, surgical techniques, and timing of tracheotomy in modern intensive care medicine.

## Lernziele

Nach Lektüre dieses Beitrags …

- können Sie die Indikationen zur Tracheotomie benennen;
- wissen Sie, wie wichtig die Bronchoskopie bei der perkutanen dilatativen Tracheotomie (pDT) ist (merke: keine dilatative Tracheotomie ohne Bronchoskopie!);
- kennen Sie jeweils geeignete Verfahren der Tracheotomie für unterschiedliche Krankheitsbilder;
- haben Sie einen Überblick über die technische Durchführung der unterschiedlichen Tracheotomieformen;
- ist Ihnen die Argumentation zum optimalen Zeitpunkt der Tracheotomie bekannt.

## Hintergrund

Die Tracheotomie wird bereits seit dem Beginn der modernen Intensivmedizin als Alternative zum translaryngealen Atemweg durchgeführt und gehört zu den häufigsten operativen Eingriffen bei Intensivpatienten [1, 2, 3]. Grundsätzlich gibt es 2 unterschiedliche Verfahren zur Durchführung einer Tracheotomie: die pDT und die chirurgische Tracheotomie (cT). Die Notfalltracheotomie bzw. -koniotomie ist nicht Thema dieses Artikels.

Trotz der **häufigen Anwendung**

häufigen Anwendung

dieser Prozedur gibt es mit Ausnahme spezieller Krankheitsbilder (z. B. hoher Querschnitt) keine einheitlich anerkannten und international akzeptierten Empfehlungen, ob, wann und mit welcher Methode eine Tracheotomie durchgeführt werden sollte. In Deutschland wird die pDT bei fehlenden Kontraindikationen allerdings zumindest in der aktuellen Leitlinie zur Beatmung favorisiert [4].

Insgesamt gibt es **vielfältige Gründe**

vielfältige Gründe,

eine Tracheotomie bei Intensivpatienten durchzuführen. Diese finden sich insbesondere bei Patienten mit akuter traumatischer oder nichttraumatischer Hirn- oder Rückenmarksschädigung, bei Patienten mit langer Beatmungsdauer und bei komplexen Verletzungen im Bereich des Gesichts oder des Kehlkopfs. Die grundsätzlichen Gedanken hinter einer Tracheotomie sind die Reduktion der Analgosedierung sowie deren Komplikationen und die Erleichterung der Spontanatmung.

## Indikationen

Vor der Indikationsstellung einer Tracheotomie ist zu klären, ob ein derartiges Prozedere im erklärten oder mutmaßlichen Sinne des Patienten ist. Auch ist es wichtig zu wissen, dass die A‑priori-Abschätzung, ob ein Patient eine **langfristige Beatmung**

langfristige Beatmung

benötigen wird, unzuverlässig sein kann (s. im Folgenden).

### Merke

Vor einer Tracheotomie ist immer zu klären, ob das Vorgehen im Sinne des Patienten ist!

Mögliche Indikationen für eine Tracheotomie sind (siehe auch Tab. 1):

- Sicherung des Atemwegs bei Atemwegsverlegung (z. B. Verletzungen, Tumor);
- Schutz vor Aspiration;
- prolongierter Entwöhnungsprozesse von invasiver Beatmung;
- Langzeitbeatmung, dauerhafte Beatmung.

**Tab. 1:** Indikationen zur Tracheotomie

	Erwartete Dauer der Beatmung	Indikation	Methode
Patient mit Atemwegsverlegung	Kurz	Atemwegssicherung	Koniotomie, chirurgische Tracheotomie
Intensivpatient	< 30 Tage	Schonung der Glottis; Verminderung des Atemtotraums/Pendelvolumens; Bronchialtoilette	Dilatative Tracheotomie
Intensivpatient	> 30 Tage	Schonung der Glottis; Verminderung des Atemtotraumes/Pendelvolumens; Bronchialtoilette	Chirurgische Tracheotomie
Patient mit neurologischen Defiziten	> 30 Tage	Vermeidung von Aspiration/via falsa	Chirurgische Tracheotomie mit mukokutaner Anastomose

Unter **prolongierter Entwöhnung**

prolongierter Entwöhnung

als Indikation zur Tracheotomie wird eine invasive Beatmung länger als 7 Tage nach dem ersten erfolglosen Spontanatmungsversuch (SBT) verstanden [5].

Da nicht bei jedem invasiv beatmeten Patienten ein SBT durchgeführt wird, ist die Indikation „prolongierte Entwöhnung“ definitionsgemäß nicht auf jeden Patienten übertragbar. In diesen Fällen kann der, allerdings unscharf definierte, Begriff „**Langzeitbeatmung**

Langzeitbeatmung

“ zur Indikationsstellung herangezogen werden. Von Langzeitbeatmung spricht man in diesem Kontext ab einer invasiven Beatmungsdauer von 2–3 Wochen [4].

Eine Tracheotomie bei prolongiertem Entwöhnungsprozess oder Langzeitbeatmung erfolgt mit dem Ziel, die negativen Auswirkungen einer fortgesetzten Beatmung über einen Endotrachealtubus zu minimieren und die Aussicht auf eine **erfolgreiche Entwöhnung**

erfolgreiche Entwöhnung

zu verbessern.

Vorteile der Tracheotomie sind u. a.:

- reduzierter Sedierungsbedarf mit dem Ziel eines wachen, kooperativen und mobilisierbaren Patienten;
- verminderte Atemarbeit durch Reduktion des Totraumvolumens und des Atemwegswiderstands;
- variable Anwendung von kontrollierten und Spontanatmungsverfahren bei gesichertem Atemweg;
- Entlastung gefährdeter anatomischer Strukturen vor allem im Larynxbereich;
- Reduktion des Risikos für Atemwegsinfektionen;
- verbesserte Mundpflege;
- Kommunikationsmöglichkeit via Sprechventil;
- erhöhter Patientenkomfort.

## Methoden der Tracheotomie

Die Tracheotomie ist ein Eingriff der grundsätzlich auf der Intensivstation durchgeführt werden kann. Die pDT ist dabei heutzutage die am häufigsten durchgeführte Methode bei Intensivpatienten. Gründe dafür sind u. a. die **flexiblere Planbarkeit**

flexiblere Planbarkeit

(Intervention durch das intensivmedizinische Team) und eine reduzierte Rate an Wundinfektionen im Vergleich mit einer chirurgischen Vorgehensweise [6]. Die **Einschrittdilatationsmethode**

Einschrittdilatationsmethode

nach Ciaglia ist die weltweit am weitesten verbreitete Methode [2]. Zwingend notwendig hierbei ist ein Vorgehen unter Sicht. Formal reicht eine Tracheoskopie aus; in der Intensivmedizin wird allerdings nahezu immer eine **Bronchoskopie**

Bronchoskopie

durchgeführt. Daher wird dieser Terminus hier auch verwendet. Als Alternative steht die cT zur Verfügung. Die Vor- und Nachteile beider Vorgehensweisen kann man Tab. 2 entnehmen. Die cT erfolgt meist mit **mukokutaner Anastomose**

mukokutaner Anastomose.

Die sog. Hybridtracheotomie vereint die Vorteile beider Techniken und wurde u. a. in unserer Klinik während der Coronapandemie vorzugsweise angewendet [7]. Auch kann eine primär dilatativ angelegte Tracheostomie sekundär chirurgisch umgewandelt werden.

**Tab. 2:** Vor- und Nachteile der perkutanen dilatativen Tracheotomie (*pDT*) und der chirurgischen Tracheotomie (*cT*)

	pDT	cT
Bronchoskopie zwingend notwendig	Ja	Nein
Infektionsrisiko [6]	Geringer	Höher
Risiko für Trachealstenose	Gleich	Gleich
Risiko für nichtsicheren Atemweg	Höher	Geringer
Risiko für Verlust des Atemwegs bei akzidenteller Dekanülierung	Höher	Geringer
Langzeitbeatmung	Eher nicht geeignet	Eher geeignet
Passagere Beatmung	Eher geeignet	Eher nicht geeignet

Die Auswahl des geeigneten Verfahrens basiert daher auf mehreren Faktoren, wie z. B.:

- den anatomischen Verhältnissen,
- dem Alter des Patienten,
- der Indikation zur Tracheotomie,
- der voraussichtlichen Dauer der Nutzung des Tracheostomas.

### Merke

Keine dilatative Tracheotomie ohne gleichzeitige Bronchoskopie!

Grundsätzlich ist Folgendes zu beachten:

1. Die Lagebeziehung der Trachea zu den umliegenden Weichteilstrukturen muss geklärt sein. Während dies bei der cT durch die operative Präparation erfolgt, ist bei den perkutanen Punktionstechniken eine präinterventionelle sonographische Würdigung der Gewebsstrukturen obligatorisch. So können Verletzungen von Gefäßen und Schilddrüse mit erheblicher Blutungsgefahr, aber auch – bei nicht mittig gelegener Trachea – die tangentiale Punktion der Trachea mit Spangenbrüchen und Risstraumata und die Verletzung des Ösophagus reduziert werden. Spangenbrüche können allerdings auch bei korrekt durchgeführten pDT auftreten.
2. Da ein Funktionsverlust der Trachea nicht mit dem Leben vereinbar ist, muss der Atemweg während der Tracheotomie über eine transorale/transnasale Intubation oder in sehr seltenen Fällen durch ein starres Beatmungstracheoskop [8, 9] gesichert sein.
3. Die Trachea sollte möglichst nicht oberhalb der zweiten Trachealspange mittig eröffnet werden; höhere Zugänge in die Trachea bergen die Gefahr der Verletzung des Ligamentum conicum, des Ligamentum cricotracheale und des Ringknorpels und damit einer späteren aufwändig zu therapierenden Larynx- oder Trachealstenose. Dabei ist erwähnenswert, dass es auch in diesem Bereich gelegentlich nicht komplett ausgeprägte, oder auch miteinander anastomosierende Trachealspangen gibt. Auch zu tiefe Punktionen können allerdings zu Komplikationen, insbesondere Blutungen, führen.
4. Unabhängig von der Interventionstechnik sollte die Tracheotomie knorpelsparend durchgeführt werden. Jeder Verlust von Trachealspangenknorpel vergrößert die Gefahr einer späteren Trachealstenose.
5. Ist eine längerdauernde Beatmung abzusehen, sollte ein stabiles Tracheostoma vorliegen. Daher wird der cT mit mukokutaner Anastomose häufig der Vorzug gegeben, um Problemen beim Kanülenwechsel (via falsa, Verlust des Atemwegs) und chronischen Granulationen am Tracheostoma vorzubeugen. Selbstverständlich kann auch ein lang angelegtes dilatatives Tracheostoma den Anforderungen an ein stabiles Tracheostoma genügen.
6. Schließlich ist auch die lokale Expertise des Behandlungsteams ein entscheidender Punkt in der Auswahl des Verfahrens

Während bei der cT mit mukokutaner Anastomose eine sichere und persistierende Verbindung zwischen Halshaut und Trachealschleimhaut geschaffen wird, entsteht bei der cT ohne mukokutane Anastomose und bei der pDT ein zunächst **nichtepithelisierter Kanal**

nichtepithelisierter Kanal

zwischen Halshaut und Trachealschleimhaut. Die mukokutane Anastomose ist bei der Umintubation sicher, schrumpft kaum, neigt nicht zu Granulationen, muss aber in der Regel chirurgisch verschlossen werden. Das Tracheostoma ohne mukokutane Anastomose neigt zur Bildung von Granulationen, schützt insbesondere in der frühen (erste 7–14 Tage) Phase nicht vor **via falsa**

via falsa

bei Umintubation, schrumpft schnell und verschließt sich spontan (Tab. 2 und 3).

**Tab. 3:** Tracheotomie mit und ohne mukokutane Anastomose

	Tracheostoma mit mukokutaner Anastomose	Tracheostoma ohne mukokutane Anastomose
Durchführung	Chirurgisch	Chirurgisch/perkutan
Verbindung Halshaut/Trachea	Epithelisiert	Nichtepithelisiert
Granulationen	–	+
Via falsa	–	+
Schrumpfung	–	+
Verschluss	Chirurgisch	Spontan
Trachealstenose	+	+

### Merke

Vor einer dilatativen Tracheotomie ist immer eine sonographische Darstellung der Halsweichteile durchzuführen.

## Risiken

Das *operative* Risiko ist bei einer cT naturgemäß geringer als bei einem dilatativen Vorgehen, da der Chirurg unter Sicht arbeitet. Bei der dilatativen Tracheotomie sind **Blutungen**

Blutungen,

die Verletzung der Trachealhinterwand sowie tracheoösophagiale oder tracheokutane Fisteln möglich und aufklärungspflichtig. Insgesamt hat die pDT dennoch ein günstiges Komplikationsrisiko (weniger Infektionen, weniger Vernarbungen). Schwerwiegende Komplikationen treten bei beiden Verfahren ungefähr gleich häufig auf [10].

Absolute Kontraindikationen gegen eine Tracheotomie im Allgemeinen existieren nicht. Allerdings sollten die Risiken eines solchen Eingriffs gegen den potenziellen Nutzen abgewogen werden.

In folgenden Situationen sollte eine pDT nicht durchgeführt werden:

1. schwieriger Atemweg;
2. Sonographie der Halsweichteile nicht möglich;
3. interventionelle Endoskopie der Trachea nicht gewährleistet.

Bei **unsicherem Atemweg**

unsicherem Atemweg

bzw. fehlender Intubationsmöglichkeit (z. B. bei extremer Kyphose der Halswirbelsäule oder im Fall der akuten Verlegung der oberen Atemwege) besteht bei frustraner Punktion keine weitere Möglichkeit der Beatmung. Es ist in diesem Fall notwendig, die Trachea von vorn herein chirurgisch darzustellen. Dies gilt in viel stärkerem Maß auch für die **akzidentelle Dekanülierung**

akzidentelle Dekanülierung.

Hier kann der Verlust des Atemwegs lebensbedrohend sein.

Die pDT darf nur nach sonographischer Darstellung der Halsweichteile und mit gleichzeitiger Trachealendoskopie durchgeführt werden. Dies ist wichtig, um die direkte Lagebeziehung zu größeren Blutgefäßen darzustellen und die Verletzung dieser Gefäße mit der Gefahr einer massiven Blutung zu vermeiden.

## Bestimmung des optimalen Zeitpunkts: früh vs. spät

Gemeinhin werden Tracheotomien, die in der ersten Woche der invasiven Beatmung durchgeführt werden, als „frühe“ und solche nach den ersten 7 Tagen als „späte“ Tracheotomien bezeichnet. Interessanterweise ist der ideale Zeitpunkt für eine Tracheotomie auch heute noch weitgehend unklar [11, 12]. Prospektive randomisierte Studien mit großen Fallzahlen, die eine frühe mit einer späten Tracheotomie vergleichen, ergaben unterschiedliche Ergebnisse.

### Aktuelle Studienlage

In einer **Metaanalyse**

Metaanalyse

aus dem Jahr 2022 wurden insgesamt 19 Studien mit teils kleinen Patientenkollektiven untersucht [13].

In einer Cochrane-Analyse [11] aus dem Jahr 2015 wurde mit moderater Evidenz gezeigt, dass eine frühe Tracheotomie mit einer niedrigeren Mortalität assoziiert zu sein scheint. Allerdings verweisen die Autoren auf eine sehr **hohe Inhomogenität**

hohe Inhomogenität

und Inkonsistenz bei den einzelnen Arbeiten. Im Wesentlichen zeigt nur eine Arbeit aus dem Jahr 2004 an internistisch schwer vorerkrankten Patienten eine geringere Mortalität [14], während dies durch die anderen Untersuchungen nicht nachzuweisen war. Insgesamt schlussfolgern die Autoren, dass ein Überlebensvorteil für eine frühe Tracheotomie nicht gezeigt werden konnte [11].

Die bereits erwähnte aktuelle Metaanalyse [13] konkretisiert diese Ergebnisse mittels Bayes-Statistik dahingehend, dass eine Reduktion der **Mortalität**

Mortalität

in geringem Ausmaß zu erwarten sei („number needed to treat“ = 100). Bei dieser Untersuchung wurden Studien aus den Jahren 1990 bis 2022 mit Patientenzahlen von 19–451 pro Arm eingeschlossen. Das Setting war auch in dieser Analyse sehr unterschiedlich (medizinisch, traumatologisch, chirurgisch, neurochirurgisch) und die Patienten sehr unterschiedlich schwer erkrankt, sodass verallgemeinernde Schlussfolgerungen schwierig zu ziehen sind.

Die Daten zur schnelleren Entwöhnung vom Respirator sind ebenfalls nicht konsistent. Weitere Outcomeparameter, wie die Pneumonierate oder die Aufenthaltsdauer auf der Intensivstation, scheinen bei der frühen Tracheotomie günstiger als bei der späten, wobei auch hier auf die Heterogenität der Daten hingewiesen wird [11, 13, 15].

#### Schlaganfall bzw. traumatische Hirnschädigung

In der **SETPOINT2-Studie**

SETPOINT2-Studie

[16], in der 382 Patienten mit einem großen Schlaganfall entweder in die Gruppe der frühen Tracheotomie oder die Standardtherapie randomisiert wurden, ließ sich ein Outcomeunterschied nicht zeigen. Als Outcome wurde hier das funktionelle Ergebnis (modifizierter Ranking-Skala-Score) nach 6 Monaten gewählt. Nach dieser Zeit betrug der Score von 0–4 (positives funktionelles Outcome) in der Frühe-Tracheotomie-Gruppe 44 % und in der Späte-Tracheotomie-Gruppe 47 %. Dieser Unterschied war bei einer Odds-Ratio (OR) von 0,93 (95 %-Konfidenzintervall [95 %-KI] 0,6–1,42) nicht signifikant [11]. Auch das Überleben auf der Intensivstation und nach 6 Monaten war nicht unterschiedlich. In einer aktuellen großen Metaanalyse mit über 17.000 kritisch kranken Patienten mit einem Schlaganfall ergaben sich ebenfalls keine Hinweise für ein besseres Überleben unter einer frühen Tracheotomie [17].

Bei Patienten mit **traumatischer Hirnschädigung**

traumatischer Hirnschädigung

(*n* = 1358) wurden in einer großen Datenanalyse [18] einer multinationalen Beobachtungsstudie die Tracheotomierate, der Zeitpunkt der Tracheotomie sowie das Outcome der Patienten untersucht. Patienten wurden in eine Gruppe mir früher Tracheotomie (< 7 Tage, etwa 40 % der Patienten) bzw. mit später Tracheotomie (> 7 Tage, etwa 60 % der Patienten) eingeteilt. Etwa ein Drittel der Patienten wurde tracheotomiert. Der Zeitraum bis zur Tracheotomie betrug bei allen Patienten im Median 9 Tage. In der univariaten Analyse wurde für die Gruppe mit früher Tracheotomie weder ein Überlebensvorteil noch ein besseres Outcome (nach 6 Monaten) gesehen. Allerdings zeigte sich in einigen Modellen der Regressionsanalyse, dass eine späte Tracheotomie mit einem schlechteren **neurologischen Outcome**

neurologischen Outcome

und Überleben assoziiert war (6 % Mortalitätssteigerung; Hazard-Ratio [HR] 1,06, 95 %-KI 1,03–1,08; *p* = 0,001). Die **Aufenthaltsdauer**

Aufenthaltsdauer

auf der Intensivstation war bei früher Tracheotomie signifikant kürzer (20 vs. 27 Tage; *p* < 0,001; [18]). Ob es sich hierbei um eine Assoziation oder Kausalität handelt, kann aufgrund des Studiendesigns nicht abschließend beurteilt werden. Ein **Konsensuspapier**

Konsensuspapier

zu Empfehlungen der Beatmungstherapie bei akuter Hirnschädigung [19] kommt schließlich zu dem Fazit, dass ein optimaler Zeitpunkt für diese Patienten nicht generell genannt werden kann.

#### Pulmonale Erkrankungen

In einer randomisierten multizentrischen Studie [20], der sog. **TracMan Study**

TracMan Study,

wurden 909 Patienten mit führend pulmonalen Erkrankungen untersucht. Der primäre Endpunkt (**30-Tage-Mortalität**

30-Tage-Mortalität)

war bei diesen Patienten nicht signifikant unterschiedlich (30,8 % in der frühen Gruppe vs. 31,5 % in der späten Gruppe). In dieser Studie wurden relativ schwer kranke Intensivpatienten (mittlerer Akutphysiologie-und-Chronic-Health-Disease-Classification-System[APACHE]-I-Wert 20) untersucht. Etwa 80 % dieser Patienten waren internistisch. Die zugrunde liegende Erkrankung für die Notwendigkeit der Tracheotomie war bei etwa 70 % der Patienten pulmonal. Insofern sind die Patientencharakteristika dieser Studienpatienten auf das Patientengut einer Intensivstation gut übertragbar.

Grundsätzlich sollte das **Weaning**

Weaning

vom Respirator auch bei Patienten mit pulmonalen Erkrankungen so schnell wie möglich erfolgen. Die „readiness to wean“ muss daher täglich geprüft werden. Sollte eine Extubation als möglich erachtet werden, ist ein Spontanatmungsversuch angezeigt. Bei einmaligem (oder auch mehrmaligem) Versagen der Extubation sollte eine Tracheotomie erwogen werden. Hierbei ist es wichtig, die möglichen Vorteile einer Tracheotomie (Reduktion der Analgosedierung, Mundhygiene, leichtere Spontanatemversuche, Reduktion des Totraums, Mobilisierung) gegen die Risiken (Blutung, Infektionen, Atemwegsverletzungen, Trachealstenosen) abzuwägen. Es wird allerdings davon ausgegangen, dass die Komplikationsrate niedrig ist [21].

##### Merke

Vor der Tracheotomie sollte die respiratorische Situation stabil sein.

Auch ist es wichtig zu wissen, dass natürlich die zugrunde liegende Erkrankung durch die Tracheotomie an sich nicht verändert wird. Allenfalls der Patientenkomfort kann verbessert werden. Schließlich müssen auch ethische Fragen zur Tracheotomie, wie z. B. eine mögliche Langzeitbeatmung, mit dem Patienten bzw. seinen Angehörigen besprochen werden. Auch sollte nicht unerwähnt bleiben, dass eine frühe Extubation und nichtinvasive Beatmung bei Patienten mit fortgeschrittener **chronischer Lungenerkrankung**

chronischer Lungenerkrankung

(COPD) eine sinnvolle Therapieoption ist, die man den Patienten mit einer frühen Tracheotomie vorenthalten würde. Schon aufgrund dieser Ausführungen ist es ersichtlich, dass es eine generelle Empfehlung für eine frühe oder späte Tracheotomie bei COPD-Patienten nicht geben kann und die Entscheidung zur Tracheotomie im Team getroffen werden sollte.

Dies spiegelt sich in den Empfehlungen nationaler und internationaler Fachgesellschaften wider [4]. Die aktuelle deutsche S3 Leitlinie empfiehlt daher im Sinne einer Expertenempfehlung, bei invasiv beatmeten Patienten mit nichtabsehbarer Beatmungsdauer die Durchführung einer Tracheotomie. Eine **Frühtracheotomie**

Frühtracheotomie

wird allerdings nicht empfohlen [4].

Wichtig ist auch zu erwähnen, dass eine frühzeitige Tracheotomie innerhalb der ersten 7 Tage nach Intubation erst dann erfolgen sollte, wenn sich die **Beatmungsparameter**

Beatmungsparameter

insoweit stabilisiert haben, dass sich eine Tracheotomie mit den prozedurimmanenten Risiken (Apnoe, Verlust des positiven endexpiratorischen Drucks [PEEP], Relaxation) gefahrlos durchführen lässt.

### Einordnung der Studienergebnisse

Erwähnenswert aus beiden zuvor genannten randomisierten Untersuchungen [16, 20] ist, dass 53 % bzw. 33 % der in die „späte“ Gruppe randomisierten Patienten keinerlei Tracheotomie erhielten. In beiden Untersuchungen waren in dieser Gruppe zwei Drittel der Patienten vor Erreichen des Tracheotomiezeitpunkts bereits erfolgreich extubiert. Demzufolge wurde in der „frühen“ Gruppe mutmaßlich eine relevante Zahl an Patienten tracheotomiert, die in den nächsten Tagen erfolgreich hätten extubiert werden können.

In beiden Studien war eines der Einschlusskriterien eine erwartete invasive Beatmungsdauer von ≥ 7 bzw. ≥ 14 Tagen. Die hohen Raten an erfolgreicher Extubation vor später Tracheotomie sind somit möglicherweise auch Ausdruck der Schwierigkeit, die voraussichtliche Beatmungsdauer selbst durch Intensivmediziner hinreichend gut abschätzen zu können.

Diese Beobachtungen weisen auf ein **Dilemma**

Dilemma

hin, das uns z. B. auch bei der Entscheidungsfindung hinsichtlich eines mechanischen Unterstützungssystems beim kardiogenen Schock begegnet: eine valide Charakterisierung, welcher Patient überhaupt und ggf. zu welchem Zeitpunkt von einer Intervention profitiert.

#### Merke

Die Entscheidung zur Tracheotomie ist patientenindividuell zu treffen.

### Resümee

Sowohl in der S3-Leitlinie „Invasive Beatmung und Einsatz extrakorporaler Verfahren bei akuter respiratorischer Insuffizienz“ als auch in der S2k-Leitlinie „Prolongiertes Weaning“ wird die Frühtracheotomie nicht empfohlen [4, 5].

Die, gerade auch aus Patientenperspektive, unstrittigen **potenziellen Vorteile**

potenziellen Vorteile

einer Tracheotomie, wie z. B. Mundpflege, Kommunikation, Mobilisation und Nahrungsaufnahme, werden durch die Prozedur an sich nur unvollständig erreicht. Insbesondere zur Wiederherstellung eines möglichst ungestörten Schluckvorgangs und Sprechvermögens ist ein intensives Training mit logopädischen, atem- und physiotherapeutischen Elementen zwingend erforderlich.

Die Entscheidung zur Tracheotomie an sich sowie der Zeitpunkt sind immer sehr individuell und patientenorientiert zu treffen. Hierbei ist der (mutmaßliche) **Patientenwille**

Patientenwille

einzuholen und die Entscheidung schließlich im Team zu treffen.

## Fazit für die Praxis

- Die Tracheotomie ist einer der häufigsten operativen Eingriffe bei Intensivpatienten.
- Es wird die perkutan dilatative von der chirurgischen Tracheotomie unterschieden. Die chirurgische Tracheotomie kann mit und ohne mukokutane Anastomose erfolgen.
- Die Hybridtracheotomie kann die Vorzüge beider Methoden miteinander vereinen.
- Im Allgemeinen wird zwischen einer frühen (<7 Tage nach Intubation) und einer späten Tracheotomie (> 7 Tage nach Intubation) unterschieden.
- Generelle Vorteile für eine frühe oder späte Tracheotomie existieren nicht.
- Die Entscheidung zur Tracheotomie sollte im Team und erst nach ausführlicher Beratung der Patienten bzw. Angehörigen erfolgen. Eine Korrektur der Grunderkrankung erfolgt durch die Tracheotomie nicht.

## CME-Fragebogen

Welche Aussage zur Tracheotomie ist richtig?

Vor einer Tracheotomie ist der potenzielle Patientenwillen zu ermitteln.

Eine chirurgische Tracheotomie und eine dilatative Tracheotomie weisen die gleichen Risiken auf.

Das Infektionsrisiko ist bei der dilatativen Tracheotomie höher als bei der chirurgischen.

Trachealstenosen kommen im Verlauf praktisch nie vor.

Die Trachealhinterwand kann eigentlich nie verletzt werden.

Welche Aussage zur dilatativen Tracheotomie ist richtig?

Es muss eine Bronchoskopie während der Intervention stattfinden.

Die sonographische Darstellung der Halsweichteile ist verzichtbar.

Sie sollte nur durchgeführt werde, wenn aus anatomischen Gründen eine chirurgische Tracheotomie nicht möglich ist.

Sie sollte bei Patienten mit schwierigem Atemweg durchgeführt werden.

Sie sollte möglichst frühzeitig (in den ersten 3 Tagen) durchgeführt werden.

Ein Patient (ischämischer Schlaganfall) wird seit 10 Tagen auf der Intensivstation beatmet, da es rezidivierend zu Aspirationspneumonien kommt. Welches Vorgehen ist zu favorisieren?

Der Patient sollte umgehend chirurgisch tracheotomiert werden, da ein Wiedererlangen der Schluckfähigkeit nahezu ausgeschlossen ist.

Der Patient sollte in frühestens 10 Tagen tracheotomiert werden, da eine frühe Tracheotomie bei Schlaganfall kontraindiziert ist.

Eine dilatative Tracheotomie ist aktuell ein probates Vorgehen, da ein Wiedererlangen der Schluckfähigkeit möglich ist.

Auf keinen Fall sollte eine chirurgische Tracheotomie erfolgen, da diese bei Schlaganfall kontraindiziert ist

Auch bei schwierigem Atemweg muss die Tracheotomie dilatativ erfolgen.

Was ist im Rahmen einer Tracheotomie bei Patienten mit schwierigem Atemweg wichtig?

Es sollte eine chirurgische Tracheotomie durchgeführt werden, um unter Sicht die Trachea freilegen zu können.

Vor einer dilatativen Tracheotomie ist eine Sonographie in jedem Fall ausreichend, um die anatomische Situation einzuschätzen.

Es sollte eine dilatative Tracheotomie durchgeführt werden, idealerweise unter Bronchoskopie.

Es sollte nach chirurgischer Tracheotomie eine Sonographie der Halsweichteile durchgeführt werden.

Eine Bronchoskopie ist bei einer dilatativen Tracheotomie nicht notwendig. Die Kanülenlage kann auch später korrigiert werden.

Ein Patient wird nach langwieriger Beatmung schließlich in ein Pflegeheim mit Beatmung verlegt. Was gilt idealerweise für das Tracheostoma?

Das Tracheostoma sollte dilatativ perkutan angelegt sein.

Vor der Verlegung in ein Pflegeheim muss das Tracheostoma verschlossen sein.

Das Tracheostoma sollte chirurgisch mit mukokutaner Anastomose angelegt sein.

Es ist egal, ob das Tracheostoma dilatativ oder chirurgisch angelegt wurde, da eine Trachelkanülendislokation ausgeschlossen ist.

In einem Pflegeheim muss die Trachealkanüle immer angenäht sein.

Was ist bei der Durchführung einer Tracheotomie zu beachten?

Bei schwieriger Anatomie, z. B. Adipositas per magna, ist die chirurgische Tracheotomie der dilatativen vorzuziehen.

Vor einer dilatativen Tracheotomie müssen die Halsweichteile nicht dargestellt werden.

Die chirurgische Tracheotomie kann nur ohne mukokutane Anastomose erfolgen.

Bei einer dilatativen Tracheotomie ist die Bronchoskopie entbehrlich.

Bei einer dilatativen Tracheotomie kann die Bronchoskopie auch nach dem Eingriff erfolgen.

Was zählt zu den Komplikationen einer Tracheotomie?

Blutungen

Bewusstseinsstörungen

Nierenversagen

Herzrhythmusstörungen

Leberversagen

Was ist bei einem Patienten mit frischer intrazerebraler Blutung hinsichtlich des optimalen Zeitpunktes zur Tracheotomie zu beachten?

Der Zeitpunkt hängt vom klinischen Zustand des Patienten sowie seinen Begleiterkrankungen ab.

Die deutsche S3-Leitlinie empfiehlt eine Frühtracheotomie innerhalb der ersten 7 Tage.

In einer randomisierten Studie konnte durch eine späte Tracheotomie eine Mortalitätsreduktion gezeigt werden.

Es ist vollkommen egal, wann eine Tracheotomie durchgeführt wird, da der klinische Zustand des Patienten unerheblich ist.

Ein Patient mit intrazerebraler Blutung sollte grundsätzlich nicht tracheotomiert werden.

Was sind relative Kontraindikationen der perkutanen Dilatationstracheotomie?

Adipositas per magna

Ausgeheilte Pneumonie

Lungenemphysem

Zustand nach Herzinfarkt

Passagere Schluckstörungen

Welche Aussage zur chirurgischen Tracheotomie (cT) im Vergleich zur dilatativen perkutanen Tracheotomie ist richtig?

Beide Verfahren können vollkommen unabhängig von der Indikation eingesetzt werden.

Die chirurgische Tracheotomie darf nur bei Patienten mit Langzeitbeatmung durchgeführt werden.

Bei beiden Verfahren sollte der Zugang grundsätzlich unterhalb der 2. Trachealspange erfolgen, um subglottische Stenosen zu vermeiden.

Nach einer dilatativen Tracheotomie muss analog zur cT der definitive Verschluss chirurgisch erfolgen.

Die dilatative Tracheotomie darf nicht bei Patienten mit Langzeitbeatmung durchgeführt werden.
